# Editorial: Horizons of autism spectrum disorder and attention deficit hyperactivity disorder in clinical practice

**DOI:** 10.3389/fpsyt.2023.1168785

**Published:** 2023-04-04

**Authors:** Fei Li, Yiping Shen, Tingyu Li

**Affiliations:** ^1^Developmental and Behavioral Pediatric Department and Child Primary Care Department, Brain and Behavioral Research Unit of Shanghai Institute for Pediatric Research, MOE- Shanghai Key Laboratory for Children's Environmental Health, Xinhua Hospital, Shanghai Jiao Tong University School of Medicine, Shanghai, China; ^2^Division of Genetics and Genomics, Boston Children's Hospital, Boston, MA, United States; ^3^Department of Neurology, Harvard Medical School, Boston, MA, United States; ^4^Ministry of Education Key Laboratory of Child Development and Disorders, Chongqing Key Laboratory of Childhood Nutrition and Health, National Clinical Research Center for Child Health and Disorders, Children Nutrition Research Center, Children's Hospital of Chongqing Medical University, Chongqing, China

**Keywords:** autism spectrum disorder, attention deficit hyperactivity disorder, risk factor, management, clinical practice

## Introduction

Autism spectrum disorder (ASD) and attention deficit hyperactivity disorder (ADHD) are two types of developmental disorders that have gained significant attention in the field of developmental and behavioral pediatrics. However, in clinical practice, the etiology of these disorders is under investigation and their management is still challenging. The current topic, therefore, aims to focus on the horizons of the two developmental disorders in clinical practice (1, 2). So far, nine papers have been published on this topic, of which seven are mostly about ASD studies.

## Early identification and screening for ASD

Early identification and screening for ASD are crucial for improving the prognosis of autistic children through evidence-based early intervention (3). The early clinical symptoms of ASD can be typically manifested as dysfunctions in eye contact, pointing by forefinger, response to name calling, communication, and inappropriate object use or abnormal sensory or perceptual in toddlers. However, most children (≥80%) who are diagnosed with ASD after a comprehensive evaluation at <3 years have retained their diagnosis (4). According to Wang T. et al., children with ASD are introduced to complementary foods later in their early developmental stage, which is associated with later feeding problems. Compared to typically developing controls, children with ASD also experience more feeding problems that can be linked to core symptoms of the disorder. Pediatricians should therefore advise parents to watch for difficulties in adjusting to the introduction of complementary foods, as this may indicate a higher risk of feeding problems later in life and atypical social interaction and communication development. Chen et al. have also found that the communication warning behavior sub-scale of the children neuropsychological and behavioral scale-revision 2016, a commonly used developmental assessment tool for children aged 0–6 years in China, can be used to screen for ASD. A communication warning behavior score that is 12 points or more above the norm requires further attention and comprehensive diagnostic evaluation to achieve early detection and diagnosis of ASD in children.

## Possible biomarker for ASD

ASD diagnosis has been made according to ASD criteria of DSM-5, which are mainly describing developmental milestones in areas of social interaction, communication, restricted interest, and stereotyped behaviors. Biomarkers for ASD diagnosis are desirable and are the topic for extensively research. Many ASD-related genes are found in subjects with ASD, especially for syndromic autism (5). In their research, Zhang et al. have observed a significant increase in sialidase NEU1 mRNA levels in children with autism, and have also found a correlation between increased NEU1 expression and social dysfunction. These findings suggest the need for further investigation into the relationship between NEU1 and ASD. Maternal low vitamin D status is regarded as a possible risk factor for ASD in offspring. Serum hypo vitamin D is common and has been recommended as a possible biomarker for ASD diagnosis (6). Several studies have reported that vitamin D supplementation is useful in improving the autistic symptoms. Shan et al. have discovered that the vitamin D levels in children with ASD are linked to electronic screen time, age, and duration of exposure to sunlight. These findings suggest that regulating screen time could be an alternative approach to managing the vitamin D nutritional status of children with ASD.

## Effect of the Early Start Denver Model (ESDM) on children with ASD is related to different traditional Chinese medicine type

Early intervention of ASD has a significant effect on the rehabilitation of ASD. Although the effectiveness of the Early Start Denver Model (ESDM) for treating ASD has been established, there remains significant variability in treatment response among individuals. As per a prior study, a collection of social cognitive skills such as receptive and expressive language, intention to communicate, and attention to faces, have consistently been associated with response to ESDM (7). It is rare to explore the application of Chinese traditional medicine in the treatment of Children with ASD. In their research, Wang L. et al. examined the relationship between three different types of children with ASD, as classified by traditional Chinese medicine (kidney jing deficiency, liver qi stagnation, and deficiency of both the heart and spleen), and the effectiveness of the Early Start Denver Model (ESDM) treatment. The authors discovered that ESDM was effective in treating all three types of children with ASD, with the group experiencing liver qi stagnation exhibiting the most notable improvements. A multi-center prospective investigation of outcome of behavioral intervention on the different traditional Chinese medicine types of ASD may be useful to give strong evidence to predict the effect of the behavioral intervention.

## Screen time in early life is a possible environmental risk factor for ADHD

The etiology of ADHD is still uncertain now, however, genetic conditions and many environmental factors are involved in the pathogenic process. Excessive screen exposure time is adverse to children's health (8). According to the research conducted by Wu et al., there could be a link between early exposure to screens and hyperactive behaviors in children. The study revealed that more than 90 minutes of screen time per day in children under the age of 3 was associated with hyperactive behaviors. These findings suggest that restricting screen time in toddlers may prove advantageous in preventing ADHD.

## Nonpharmacological intervention for ADHD management

Mainstreams of ADHD management include medication and non-medication therapy. Medications including stimulants (methylphenidate, amphetamine, etc.) and non-stimulants (atomoxetine, clonidine, etc.) are usually used in ADHD children over 6 years (9). However, in clinic many ADHD children over 6 years managed under medication or not, and most of ADHD children under 6 years old still need non-medication management.

In their study, Chu et al. investigated the impact of combining group executive functioning and online parent training on school-aged children (between 6 to 8 years old) diagnosed with ADHD. Their findings suggest that this combination approach holds potential as a non-pharmaceutical therapeutic option for younger students with ADHD. Wang Y-c et al. have designed the scheme that effects of high-definition transcranial direct current stimulation (HD-tDCS) on the right orbital frontal cortex in the treatment of ADHD. This study draws cautious conclusions that HD-tDCS leads to significant improvements in cognitive measures of attention maintaining.

## Conclusions

The current work explores the basic practice in clinics for ASD and ADHD. These results support that early behavioral study for recognition, diagnosis and early intervention for ASD are crucial to improve the clinical symptoms, limiting screening time in toddlers may be beneficial for ADHD prevention in toddlers and nutritional vitamin D status in children with ASD, and non-medication therapy is still important in clinical practice. More studies based on clinical practice would be valuable in guiding treatment decisions and improving the prognosis of ASD and ADHD.

## Author contributions

All authors listed have made a substantial, direct, and intellectual contribution to the work and approved it for publication.
